# A Transition Structure from Stripline to Substrate-Integrated Waveguide Based on LTCC

**DOI:** 10.3390/mi17020155

**Published:** 2026-01-26

**Authors:** Lu Teng, You Zhou, Ting Zhang, Zhongjun Yu, Shunli Han

**Affiliations:** 1 Science and Technology on Electronic Test & Measurement Laboratory, The 41st Institute of CETC, Qingdao 266000, China; tenglu@ei41.com (L.T.);; 2 Aerospace Information Research Institute, Chinese Academy of Sciences, Beijing 100094, China

**Keywords:** substrate integrated waveguide, transition structure, stripline, low temperature co-fired ceramics (LTCC)

## Abstract

With the advancement of wireless communication technologies into high-frequency millimeter wave and sub-THz bands, conventional transmission lines such as microstrip and stripline face significant limitations. Under the circumstances, along with the increased application of new transmission lines such as substrate-integrated waveguides (SIWs), the design of transition structures between different transmission lines has become a practical requirement in modern signal transmission systems. This paper presents a novel stripline to SIW transition structure. Drawing inspiration from the classical microstrip probe techniques in metal waveguides, the proposed design employs Low-Temperature Co-fired Ceramic (LTCC) technology for both device fabrication and SIW implementation. The developed structure demonstrates a stable performance, structural simplicity, and manufacturing feasibility. Through fabrication and testing, the transition structure can achieve a return loss below −10 dB across the 89–100 GHz frequency range, with an insertion loss of approximately 0.75 dB.

## 1. Introduction

With the continuous development of wireless communication technology, the frequency of wireless transceiver systems is gradually expanding to higher frequency bands. Traditional transmission lines such as microstrip lines and stripline exhibit significantly increased losses and more pronounced parasitic effects in high-frequency bands, making them unsuitable as the main transmission line form in the system [1,2,3,4]. Consequently, the substrate-integrated waveguide (SIW), a waveguide structure fabricated in a dielectric substrate, which offers advantages such as ease of integration, low losses, and high power capacity, has found wide applications in millimeter wave and terahertz frequency bands [4,5,6,7]. Therefore, the transition structure between an SIW and traditional transmission lines is a key technology in high-frequency communication systems [7,8,9], being especially important in highly integrated planar antenna arrays using multilayer board technology such as Low-Temperature Co-fired Ceramics (LTCC) and Printed Circuit Boards (PCBs) [10,11,12,13,14]. While existing research has predominantly focused on microstrip to SIW transitions, studies on stripline to SIW transition structures remain limited, with currently reported designs operating at lower frequency bands, exhibiting constrained transmission bandwidths, non-negligible insertion losses, and inadequate adaptability to specialized application scenarios [7,8,9]. In light of this research landscape, this paper conducts a comprehensive investigation into stripline to SIW transition structures, aiming to develop a novel transition configuration characterized by structural simplicity, operational stability, and superior transmission performance with enhanced bandwidth capabilities.

**Figure 1:**
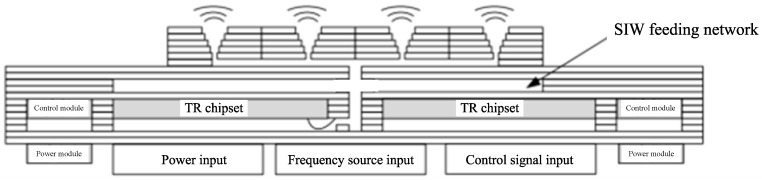
A typical antenna system architecture in multilayer board process.

Specifically, in the antenna system architecture shown in Figure 1, the W-band (75–110 GHz) operational requirements necessitate the adoption of substrate-integrated waveguide technology for the feeding network—a strategic selection that not only circumvents the excessive transmission losses inherent in conventional microstrip lines but also enables direct interfacing with substrate-integrated radiating elements, including cavity-backed (substrate-integrated cavity, SIC) and horn-type (substrate-integrated horn, SIH) antenna configurations. The transceiver chipsets are embedded inside the cavity of multilayer substrates, where their signal pins can be interconnected through two distinct methodologies: (i) flip-chip bonding directly to metallized vias that establish quasi-coaxial transmission structures that are internally connected to interlayer striplines within the substrate; (ii) conventional gold wire bonding to microstrip pads, followed by in-plane transitions that convert the microstrip lines into low-loss striplines while entering the cavity sidewalls. Consequently, the transition structure between the stripline and substrate-integrated waveguide components constituting the feeding network emerges as a critical design element for ensuring signal integrity. This constitutes the primary application context for the transition structure developed in this work.

## 2. Simulation Model Design and Optimization

### 2.1. Design of the Stripline to Substrate-Integrated Waveguide Transition Structure

**Figure 2:**
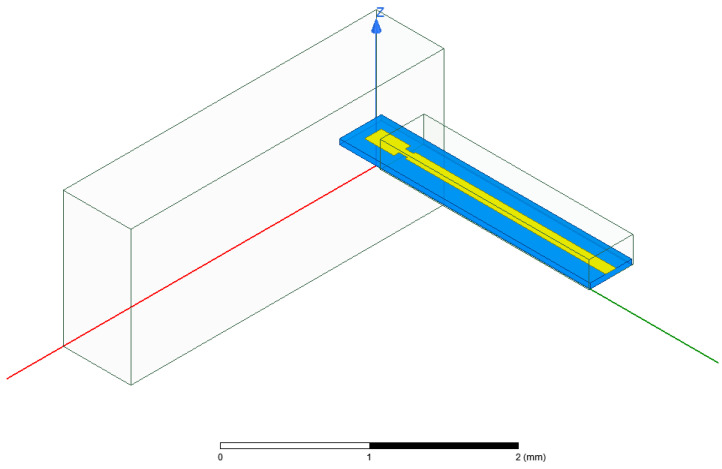
The microstrip probe transition structure.

The stripline to substrate-integrated waveguide transition structure designed in this paper is inspired by the classic microstrip probe transition structure, which is composed of a microstrip line inserted into a metal waveguide, as shown in Figure 2. More specifically, a rectangular window of appropriate size is created on the side wall of the metal waveguide, and the microstrip line substrate extends into the window to achieve electromagnetic wave transmission from microstrip lines to the metal waveguide. The microstrip probe transition structure has many applications in high-frequency millimeter wave and terahertz frequency bands [15,16,17].

**Figure 3:**
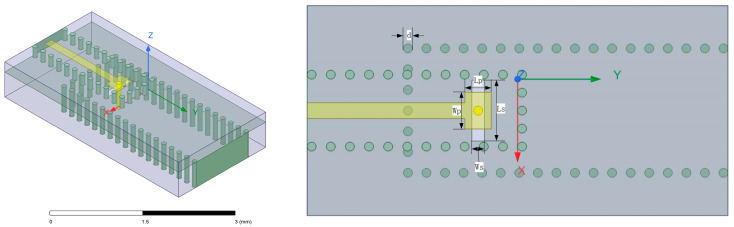
The designed stripline to SIW transition structure.

In the design of the stripline to SIW transition, the SIW is structurally analogous to the metallic rectangular waveguide mentioned above. By similarly introducing apertures on SIW sidewalls to enable probe penetration, it is found that the metal-filled vias in LTCC technology can precisely fulfill the probing function. Based on this design concept, the stripline to SIW transition structure developed in this paper has been constructed, with its corresponding simulation model illustrated in Figure 3.

In this model, the substrate of the stripline is located above the substrate-integrated waveguide substrate, with substrate thicknesses of 0.2 mm and 0.5 mm. At the end of the stripline, impedance matching is achieved through a square patch, where a vertical metal via with a depth of 0.5 mm is placed at the center of the patch as the probe inserted into the SIW. The metal via passes through a rectangular slot on the surface of the substrate-integrated waveguide, guiding electromagnetic waves into the substrate-integrated waveguide. The design dimensions of the model are as follows: the stripline width is set to 0.15 mm, the substrate-integrated waveguide width is 1.1 mm, the width *Wp* of the patch at the end of the stripline is 0.35 mm, the length *Lp* of the patch is 0.25 mm, the width *Ws* of the rectangular slot is 0.17 mm, and the length *Ls* is 0.58 mm. Additionally, the diameter *d* of the metal vias set in this LTCC process is 0.085 mm, the via pitch is set to the minimum process-allowed value—twice the via diameter, measuring 0.17 mm. The ceramic green tape material is Ferro A6M, reference dielectric constant ε_r_ = 6.21 at 95 GHz. Critical to this model’s implementation is the constraint on via depth imposed by the LTCC fabrication methodology. Since the substrate is constructed through laminating multiple uncured ceramic green tape layers, with vias mechanically drilled and metallized individually on each monolayer prior to stacking, the manufactured via depth is fundamentally discretized in increments of the single-layer green tape thickness—specifically multiples of 0.1 mm in this design.

**Figure 4:**
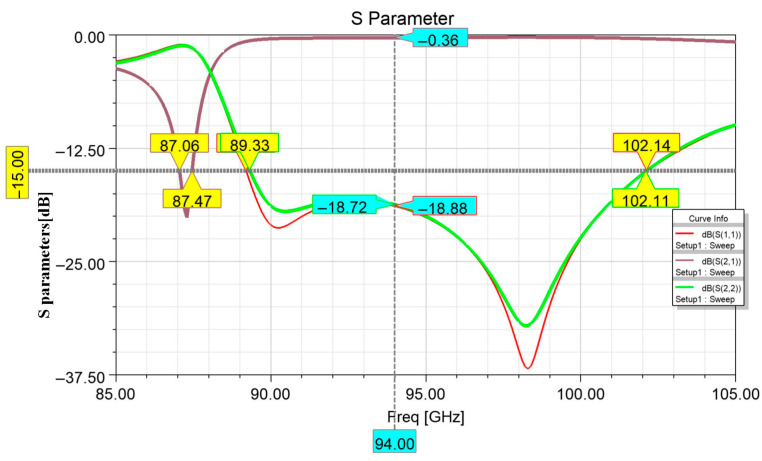
Simulation results of the stripline to SIW transition structure.

The simulation results of the stripline to SIW transition model are shown in Figure 4. From the simulation results, it can be observed that within the frequency range of 89.4–102.1 GHz, the return loss (S_11_) of this transition structure is below −15 dB, and the insertion loss (S_21_) is approximately 0.4 dB. Simulation results confirm that the proposed stripline to SIW transition structure achieves both broad operational bandwidth and excellent transmission characteristics, demonstrating strong practical viability for millimeter wave applications.

### 2.2. Design of the Substrate-Integrated Waveguide to Rectangular Waveguide Transition Structure

The proposed stripline to SIW transition structure shows promising simulation results, but its practicality still requires validation through actual fabrication, assembly, and testing. However, current millimeter wave and terahertz communication systems, along with their corresponding test instrumentation interfaces, predominantly employ standardized metallic rectangular waveguides (RWGs). For the W-band application, the WR-10 waveguide with an aperture dimension of 2.54 mm × 1.27 mm and UG-387/U flange configuration serves as the industry-standard interface. When testing planar circuits fabricated on LTCC substrates, the foremost technical challenge lies in establishing robust interconnections between the substrate and the RWG interfaces. To address this critical interfacing requirement, this study proposes an innovative transition structure that effectively bridges substrate-integrated waveguides and conventional rectangular waveguides.

The subsequent design involves a vertical transition structure that connects a rectangular waveguide propagating in the vertical direction to a substrate-integrated waveguide operating in the horizontal direction. The specific interconnection configuration is as follows: one end of the rectangular waveguide interfaces with the bottom surface of the SIW, where a window is opened in the SIW’s bottom metal layer to allow electromagnetic waves from the rectangular waveguide to couple into the SIW. The dimensions and geometry of this window on the SIW–rectangular waveguide interface directly influence the transition structure’s transmission characteristics. Furthermore, to mitigate performance degradation caused by abrupt changes in propagation direction and to achieve gradual impedance matching, stepped structures can be incorporated within the SIW to optimize transmission performance.

From the LTCC fabrication perspective, the design of the stepped SIW–rectangular waveguide transition structure is considered. Since the propagation modes of SIW closely resemble those of rectangular waveguides, the influence of cross-sectional thickness and width on SIW transmission characteristics similarly correlates with how rectangular waveguide dimensions affect their transmission properties. Considering the electromagnetic wave transition from vertical propagation in the rectangular waveguide to horizontal propagation within the LTCC substrate, the SIW-RWG interface can be equivalently modeled as a transitional sequence comprising: vertically propagating rectangular waveguide, dielectric-filled waveguide section, horizontally propagating SIW. Within the intermediate dielectric-filled waveguide section, the vertical thickness of this dielectric region should approximate λ_g_/4 to maximize transmission efficiency. However, given that SIW thickness must be integer multiples of the single green tape layer thickness in LTCC technology, this constraint fundamentally determines the substrate’s required layer count.

Regarding specific stepped structure design details, there are the following considerations: The dielectric-filled waveguide exhibits reduced dimensions compared with standard rectangular waveguides due to its dielectric loading. This dimensional reduction dictates that the stepped transition profile decreases in length along the direction away from the waveguide port. The maximum number of steps is constrained by the substrate layer count. Remaining design parameters, including individual step widths/lengths and interface window dimensions, require electromagnetic simulation optimization to determine optimal values.

**Figure 5:**
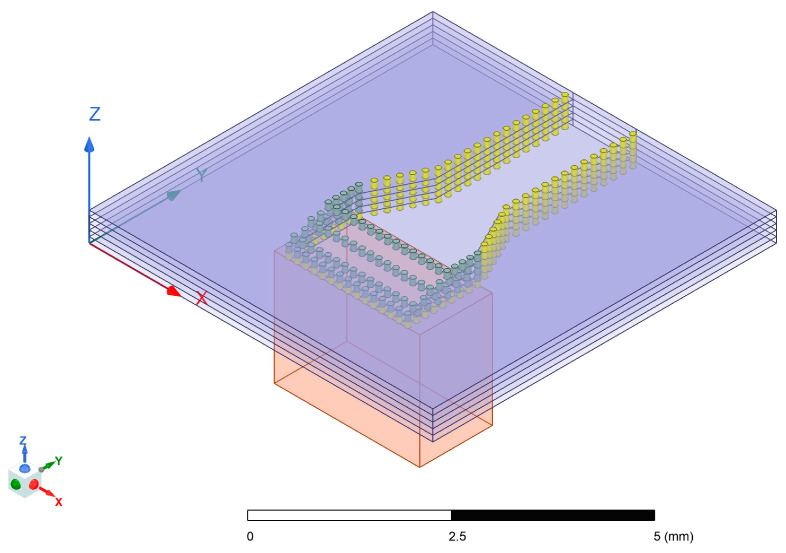
SIW-RWG stepped transition structure.

The designed transition structure from SIW to RWG is illustrated in Figure 5. This structure comprises five LTCC dielectric layers. At the transition section, progressively staggered vias between upper and lower layers form the stepped impedance transition, while printed metallization layers between each dielectric layer serve as a ground plane, electrically interconnecting the vertical metallized vias. The four-stage step lengths measure 0, 0.22 mm, 0.15 mm, and 0.4 mm. The initial dimensions of the transition-section SIW are length 1.3 mm and width 1.7 mm. Through a tapered transition segment of length 1.5 mm, this structure transitions to an ultimate SIW width of 1.1 mm. The SIW substrate’s bottom surface is coated with a metal layer. A rectangular window with dimensions of length 1.2 mm and width 1.7 mm is opened at the waveguide port interface, precisely centered on the waveguide aperture, to enable electromagnetic wave transmission. Simulation ports are assigned to the rectangular waveguide port and the LTCC substrate’s edge plane.

**Figure 6:**
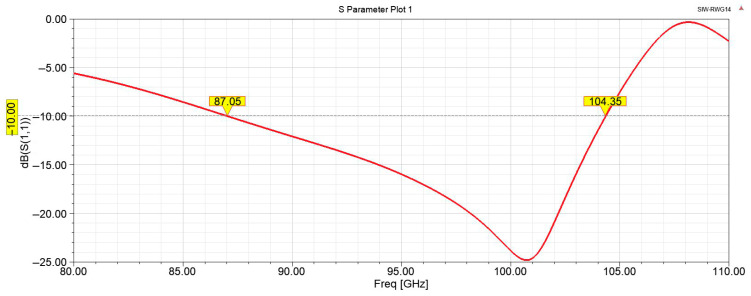
Simulation results of the SIW to RWG transition structure.

The simulation results of the SIW to RWG transition structure are presented in Figure 6. The demonstrated bandwidth sufficiently accommodates the operational requirements of the stripline to SIW transition structure, confirming its applicability for the subsequent practical fabrication and testing of stripline to SIW transitions.

### 2.3. Back-to-Back Model Design and Optimization

The proposed model shows promising simulation results, but its practicality still requires validation through actual fabrication, assembly, and testing. Therefore, it is necessary to design corresponding back-to-back structures and optimize the simulation model based on actual LTCC manufacturing processes.

**Figure 7:**
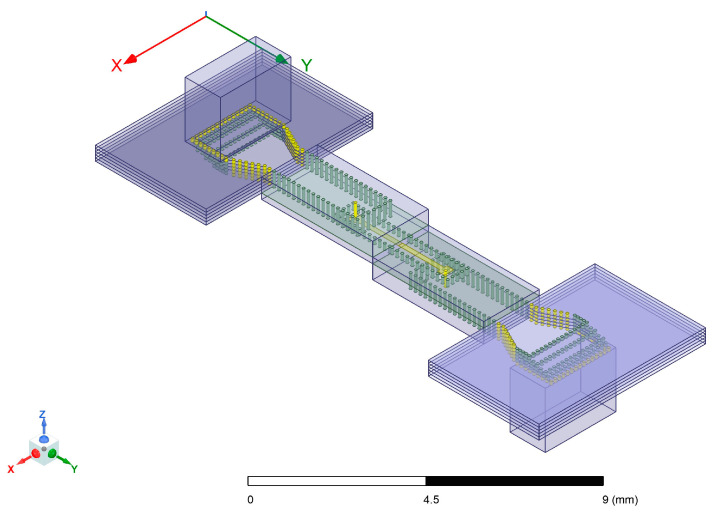
Back-to-back structure.

The back-to-back structure, as shown in Figure 7, is composed of two stripline to SIW transitions and two SIW-RWG transitions. The central stripline sections are directly interconnected, while the input/output ports of metallic waveguides are positioned on the top and bottom surfaces of the substrate. The electromagnetic wave propagation path into the model is structured as follows: metallic waveguide, RWG to SIW transition structure, SIW, SIW to stripline transition structure, stripline. The output path reverses this sequence. Consequently, when evaluating the total insertion loss of the back-to-back structure, cumulative losses from these repeated transitions must be accounted for twice. Through simulation, it has been verified that the transmission performance changes are acceptable after combining these two forms of transition structures. Detailed simulation results will be presented in subsequent figures alongside experimental measurements for comparative analysis.

Since the back-to-back model design has been completed, the simulation model can be converted into an LTCC manufacturing layout for the fabrication of the LTCC substrates. However, prior to this step, it is crucial to consider the impact of LTCC process errors and manufacturing processes on the actual transmission performance of the designed stripline to substrate-integrated waveguide transition structure.

In the series of model simulations mentioned above, the spacing between the vias arranged in rows has been set to the minimum allowed by LTCC technology, which is twice the via diameter, i.e., 0.17 mm, to achieve optimal transmission performance. Slight positioning errors among these vias will not significantly affect the overall model’s transmission performance. However, in the designed stripline to SIW transition structure model, the via acting as the probe into the SIW is particularly critical. Deviation in its position can severely impact the transmission performance of the entire model. Therefore, after the via punching process, additional check will be conducted.

**Figure 8:**
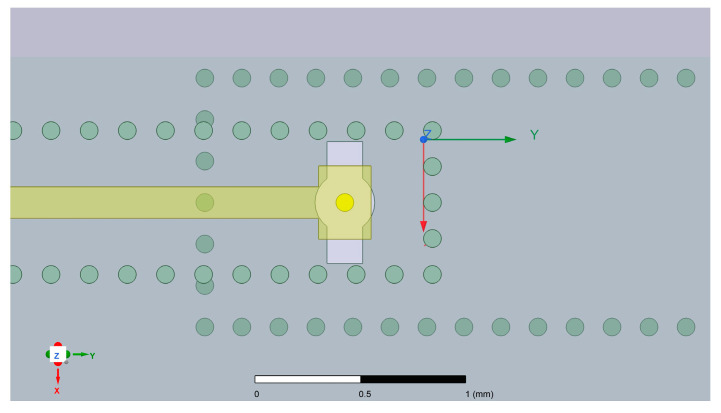
Modified slot shape.

Another concern is the distance between the probe and the edge of the rectangular slot on the upper surface of the SIW, which measures 0.0425 mm according to the design in Figure 2. Considering the positioning errors and the uncertainties caused by the overall shrinkage of the substrate during firing, it is likely to cause short circuits. Therefore, the design of the slot shape has been modified: a small circular slot overlaid on the rectangular slot around the probe position is applied, resulting in the slot shape resembling a keyhole, as depicted in Figure 8.

As for the size of the circular slot, simulation analysis has revealed that the larger the radius of the circular slot, the poorer the overall transmission performance. This is understandable because the addition of the circular slot affects the original electromagnetic wave transmission mode. However, if the radius of the circular slot is too small, it cannot effectively prevent the short circuit problem. As a result, the radius of the circular slot was set to 0.12 mm, which ensures both the overall transmission performance of the stripline to SIW transition structure and minimizes the impact of manufacturing errors.

## 3. Fabrication and Testing

**Figure 9:**
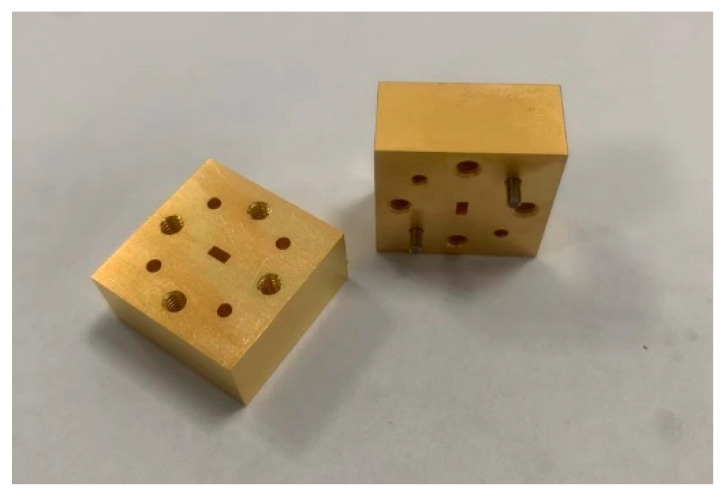
Waveguide adapters.

After completing the back-to-back model design and optimization, the next consideration is the connection between the ceramic substrate and the test instrument interface. The designed transition structure in this paper operates in the W-band frequency range, thus the test instrument interface is conforming to WR-10 standard. Therefore, two corresponding metal waveguide adapters have been designed. These adapters facilitate soldering the ceramic substrate onto the waveguide adapter and subsequently connecting it to the test instrument interface, as illustrated in Figure 9.

The dimensions of the adapter measure 20 mm × 20 mm × 10 mm, with the central waveguide interface conforming to the standard WR-10, while the flange that pairs with the waveguide port is UG-387.

**Figure 10:**
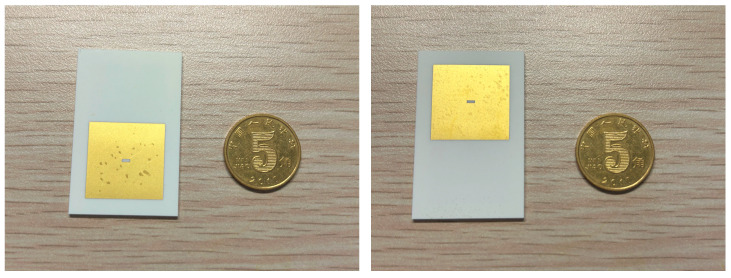
Photograph of the LTCC substrate.

To accommodate the welding dimensions of the waveguide adapters, it is necessary to add blank areas around the back-to-back structure to expand the overall substrate size. Corresponding metal pads will be placed on the upper and lower surfaces of the LTCC substrate. The final LTCC substrate for testing is shown in Figure 10.

The soldering process between the two waveguide adapters and the LTCC substrate proceeds as follows: Controlled volume of solder paste is applied to the solder pads on the substrate surface. After aligning the waveguide adapters, the substrate and the adaptor are placed together on a hot plate for heating. Once the solder paste has solidified, the process is repeated for soldering the second waveguide adapter onto the opposite side of the substrate. It is crucial to precisely control the amount of solder paste to ensure it does not spread to the waveguide aperture at the center of the solder pad. After soldering, inspection with a microscope is necessary.

**Figure 11:**
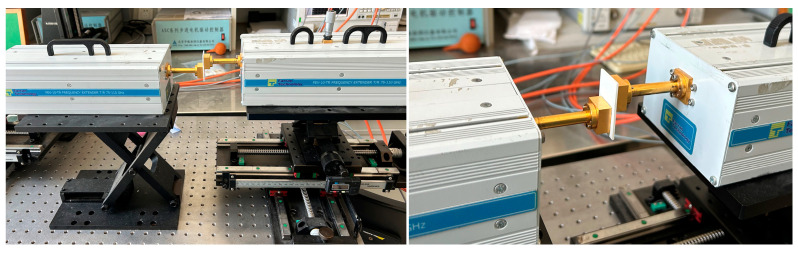
S-parameter test scenario.

Following soldering, S-parameter testing is conducted using a vector network analyzer with frequency extension modules. The frequency extender module is the Farran Technology FEV-10-TR FREQUENCY EXTENDER T/R 75–110 GHz. The test scenario is depicted in Figure 11.

## 4. Results and Discussion

**Figure 12:**
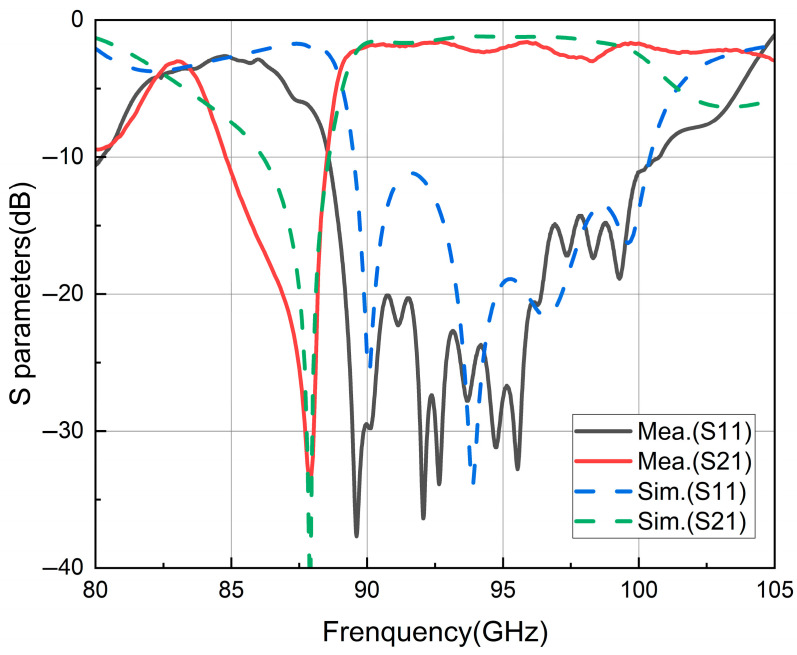
S-parameter test results.

After testing, the S-parameter data obtained are plotted as curves and shown in Figure 12, along with the simulation results of the back-to-back model.

The test results closely match the simulation results. Within the 88.6 GHz to 100.7 GHz frequency band, the return loss (S_11_) is maintained below −10 dB, ensuring good transmission performance. Simultaneously, the insertion loss (S_21_) is kept within 3 dB. Considering that the back-to-back model includes two stripline to SIW transition structures, it can be inferred that the usable bandwidth (where S_11_ remains below −10 dB) for a single stripline to SIW transition structure exceeds the frequency band from 89 GHz to 100 GHz.

Regarding insertion loss, the transition from the SIW to RWG interfaces at both ends incurs approximately 0.4 dB of loss based on the simulation. The losses for the middle stripline section and the SIW sections at both ends are estimated to be approximately 0.3 dB and 0.2 dB, respectively. Thus, the actual insertion loss for a single stripline to SIW transition structure is approximately calculated as (3 dB − 0.4 dB × 2 − 0.3 dB − 0.2 dB × 2)/2 = 0.75 dB.

Based on these test results and analysis, it is evident that the designed stripline to SIW transition structure performs effectively within the 90 GHz to 100 GHz frequency band. It achieves the initial research objectives and can be effectively utilized in signal transmission processes inside the substrate system, from transceiver chips through striplines, SIW feeding networks, to antenna front-ends.

Future improvements and applications of this design may proceed through several directions. Primarily, operational bandwidth extension can be achieved by adjusting geometric parameters such as line width and the number of SIW layers to shift the working frequency band. Secondly, the design methodology of the stripline to SIW transition could be extended to develop microstrip to SIW transition. Given the significant structural similarities between these two configurations, this adaptation requires minimal additional design effort. While the existing literature extensively addresses microstrip to SIW transitions, this approach holds potential for enhanced performance in specialized application contexts or under certain fabrication constraints. Furthermore, regarding the application scenario of this design, specifically the signal transmission from transceiver chip to SIW, the stripline was implemented to satisfy high-density wiring requirements. Also, this design approach strategically aligns with the manufacturing constraints of LTCC technology by employing the via–probe structure. Future research could continue this approach by developing direct quasi-coaxial to SIW transition, extending the quasi-coaxial structure’s central via as the probe penetrating into the SIW to simplify signal pathways and enhance transmission efficiency. Finally, unexplored refinements include implementing impedance matching through variable via diameters across layers, analogous to the microstrip width adjustments of the microstrip probe transition structure demonstrated in Figure 2. Coupled with the optimization of stripline terminal patch dimensions and SIW aperture geometry, these modifications may further enhance transmission performance while maintaining alignment with the original design framework.

## Data Availability

The datasets utilized and/or examined in this study can be obtained from the corresponding author upon making a reasonable request.

## Abbreviations

The following abbreviations are used in this manuscript:

SIW	Substrate-Integrated Waveguide
LTCC	Low-Temperature Co-fired Ceramic
PBC	Printed Circuit Board
RWGs	Rectangular Waveguides
